# Beta amyloid aggregates induce sensitised TLR4 signalling causing long-term potentiation deficit and rat neuronal cell death

**DOI:** 10.1038/s42003-020-0792-9

**Published:** 2020-02-18

**Authors:** Craig Hughes, Minee L. Choi, Jee-Hyun Yi, Seung-Chan Kim, Anna Drews, Peter St. George-Hyslop, Clare Bryant, Sonia Gandhi, Kwangwook Cho, David Klenerman

**Affiliations:** 1 Department of Chemistry, University of Cambridge, Lensfield Road, Cambridge, CB2 1EW UK; 2 Department of Clinical and Movement Neurosciences, UCL Queen Square Institute of Neurology, London, WC1N 3BG UK; 3 The Francis Crick Institute, 1 Midland Road, London, NW1 1AT UK; 4 Centre for Synaptic Plasticity, Faculty of Health Sciences, University of Bristol, Whitson Street, Bristol, BS1 3NY UK; 5 UK-Dementia Research Institute at King’s College London, King’s College, Department of Basic and Clinical Neuroscience, Institute of Psychiatry, Psychology and Neuroscience, London, SE5 9NU UK; 6 Cambridge Institute for Medical Research, University of Cambridge, Cambridge Biomedical Campus The Keith Peters Building Hills Road, Cambridge, CB2 0XY UK; 7 Department of Veterinary Medicine, University of Cambridge, Madingley Road, Cambridge, CB3 0ES UK; 8 UK Dementia Research Institute, University of Cambridge, Cambridge, CB2 0XY UK; 9 Present Address: Center for Synaptic Brain Dysfunctions, Institute for Basic Science, Daejeon, 34126 Republic of Korea; 10 Present Address: The German Center for Neurodegenerative Diseases (DZNE), Sigmund-Freud-Str. 27, Venusberg-Campus, Gebäude 99, 53127 Bonn, Germany

**Keywords:** Neuroimmunology, Alzheimer's disease

## Abstract

The molecular events causing memory loss and neuronal cell death in Alzheimer’s disease (AD) over time are still unknown. Here we found that picomolar concentrations of soluble oligomers of synthetic beta amyloid (Aβ42) aggregates incubated with BV2 cells or rat astrocytes caused a sensitised response of Toll-like receptor 4 (TLR4) with time, leading to increased production of TNF-α. Aβ aggregates caused long term potentiation (LTP) deficit in hippocampal slices and predominantly neuronal cell death in co-cultures of astrocytes and neurons, which was blocked by TLR4 antagonists. Soluble Aβ aggregates cause LTP deficit and neuronal death via an autocrine/paracrine mechanism due to TLR4 signalling. These findings suggest that the TLR4-mediated inflammatory response may be a key pathophysiological process in AD.

## Abstract (web summary)

Hughes et al. investigate the TLR4-mediated inflammatory response in Alzheimer’s disease and show that picomolar concentrations of soluble amyloid beta aggregates lead to a sensitised response of TLR4 with time, resulting in increased TNF-α production. They suggest the use of near physiological concentration of soluble aggregates can cause long-term potentiation deficit and neuronal death through an autocrine/paracrine mechanism due to TLR4 signalling.

## Introduction

Memory loss is a very common symptom of Alzheimer’s disease (AD), however the molecular basis by which memory loss occurs is not understood^1^. This means it is currently challenging to develop treatments for AD. A synaptic correlate of memory is long-term potentiation (LTP). LTP is widely considered one of the major cellular mechanisms that underlies learning and memory. It has been found that soluble beta-amyloid (Aβ) aggregates from a variety of sources including soaked brain^2^, brain homogenate, concentrated CSF and synthetic aggregates^3,4^ can cause LTP deficit in brain slices. Significant efforts have been made to identify the nature of the aggregates that affect LTP deficit, so they can be targeted for potential therapy. Antibodies that bind the N-terminus of Aβ^4^ ^4^, knock-out of PrP or the use of PrP antibodies^5^ have all been shown to be effective in preventing aggregate-induced LTP deficit. These results show that soluble Aβ aggregates initiate LTP deficit, but the mechanism by which this occurs and whether it is a result of the direct interaction of aggregates with synapses or occurs by a different mechanism has not been established to date. In vivo, Aβ can be post-translationally modified and interact with other proteins present, so that the aggregates present are heterogeneous in both size and composition. In contrast, synthetic aggregates made by aggregating Aβ42 in the test-tube are only heterogeneous in size not composition and still capable of causing LTP deficit^4^. In most experiments the aggregate concentration is not measured but only the total Aβ monomer concentration is known. This means that while it has been observed that brain-derived aggregates are more effective at causing LTP deficit than synthetic Aβ aggregates this could simply occur because the concentration of aggregates is higher in the preparations used. It is not possible to determine which type of aggregate is more effective at causing LTP without knowing the aggregate concentration.

Aβ aggregates can trigger the production of a number of proinflammatory cytokines, including TNF-α, from astrocytes and microglia^6,7^, and the media from conditioned astrocytes is toxic to neurons^8^ suggesting that neuronal cell death can occur via an inflammatory mechanism. One of the routes that pro-inflammatory cytokines are produced occurs via Toll-like receptors, pattern recognition molecules that recognize damaged molecules, particularly TLR2 and TLR4^9,10^. Our recent work shows that synthetic Aβ aggregates exist in a range of different sizes and structures with the longer protofibrils being the inflammatory species and signal via TLR4^11^. There is a crystal structure of TLR3, which is in the same family as TLR4, bound to an RNA dimer which is about 2 nm in diameter^12^. TLR3 signalling occurs when the RNA dimer is longer than 15 nm^13^. This suggests that long protofibrillar Aβ aggregates, which have a comparable diameter, initiate TLR4 signalling by forming a similar structure with a TLR4 dimers bound along the protofibril, providing a plausible explanation of both why they are the inflammatory species and how they initiate TLR4 signalling. However, to date, this experiment and many other experiments on aggregate induced inflammation have been performed at high aggregate concentrations in short time periods, typically 24 h. Therefore, there are important questions about the relevance of the results obtained at these high aggregate doses to AD. In particular, it is not clear how the response is altered at more relevant physiological concentrations of aggregates applied over longer times or if TLR4 signalling occurs at all. To address this issue, we first explored the response of BV2 microglial to extended doses of low concentrations of soluble aggregates, close to physiological levels, finding that this leads to sensitized response to these aggregates due to TLR4 signalling. We then explored if this aggregate-induced inflammatory response could lead to LTP deficit and neuronal cell death, cellular correlates of the symptoms associated with the development of AD, by performing experiments in the presence and absence of TLR4 antagonists.

## Results

### Pro-inflammatory response of macrophages to beta -amyloid

**Fig. 1:**
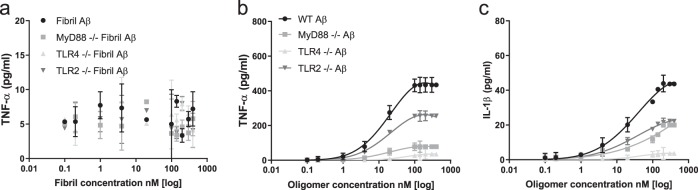
Pro-inflammatory response of TLR4, MyD88 or TLR2 knockout macrophages to Abeta42(Aβ) oligomers and fibrils. Cells (WT: wild type, MyD88^−/−^: MyD88 knockout, TLR4^−/−^: TLR4 knock out and TLR2^−/−^: TLR2 knockout) were stimulated with Aβ Fibrils (0.1–400 nM) and Aβ oligomers together with monomer (total monomer concentration 0.02–80 μM) for 24 h. The levels of the pro-inflammatory mediators TNF-α and IL-1β were measured. **a** TNF-α production remained unchanged at all concentrations with the addition of Fibrils (*n* = 5, ±sem). **b** TNF-α production significantly increases with increasing oligomer concentrations in all except the TLR4 knockout cells (*n* = 5, ±sem). **c** IL-1β levels production significantly increases with increasing oligomer concentrations in all except the TLR4 knockout cells (*n* = 5, mean ± sem).

**Fig. 2:**
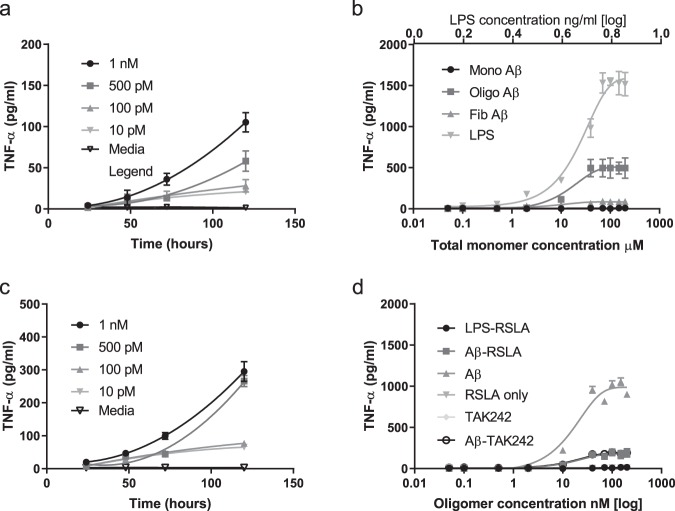
Response of BV2 microglial cells and astrocytes to soluble Abeta42 (Aβ) aggregates (oligomers) and the blocking of the TNF-α production by TLR4 antagonists. **a** Time course of TNF-α production by BV2 cells in response to continual exposure to soluble Aβ oligomers (10 pM–1 nM) together with monomer (monomer concentration 0.002–0.2 μM) (*n* = 4, sem). **b** The response of astrocytes to Aβ monomers, Aβ oligomers together with monomer (oligomer concentration 0.25 to 1000 nM and total monomer concentration 0.05–200 μM) and Aβ fibrils (total monomer concentration) compared to LPS (0.05–200 ng/ml) stimulation for 24 h (*n* = 4, mean ± sem). The astrocyte preparation had <2% microglial. **c** Time course of TNF-α production by astrocyte cells in response to continual exposure to Aβ oligomers (10 pM–1 nM) together with monomer (total monomer concentration 0.002–0.2 μM) (*n* = 4, mean ± sem). **d** The response of astrocytes after a 24 h incubation with Aβ oligomers (0.05–200 nM oligomer) together with monomer (total monomer 0.01–40 μM)) only and together with the TLR4 antagonist RSLA (0.1 µg/ml), LPS and RSLA, RSLA only and the TLR4 antagonist TAK-242 (1 µM) alone and with Aβ oligomers and monomer (*n* = 4, mean ± sem).

Experiments were firstly performed using synthetic oligomers of Aβ1-42, made by aggregating monomer and characterised by single molecule fluorescence, allowing us to estimate the soluble aggregate concentration^14,15^, and hence be able to perform reproducible experiments. These soluble aggregates, oligomers, are predominantly trimers and tetramers although they range in size from dimer to 20mers^14^ and are stable once formed allowing solutions of different initial aggregate concentrations to be made by serial dilution. This enables us to work at close to physiological concentrations of Aβ aggregates. Our initial experiments confirmed previous results that Aβ oligomers but not fibril or monomer lead to the production of TNF-α and IL-β in a BV2 microglial cell line and astrocytes in 24 h (Figs. 1 and 2). The synthetic Aβ monomer produced no TNF-α which confirmed that it contained no endotoxin contaminants. Using TLR4 and Myd88 knock-out cell lines we then showed that signalling was predominantly mediated by TLR4 and Myd88 (Fig. 1). We then performed experiments at lower aggregate concentrations for several days, close to the physiological concentration of 1–10 pM^16^. Control experiments showed that there was no significant change in the number of aggregates during the 24 hours’ incubation with cells, before buffer exchange, for total monomer concentrations below about 10 nM which is estimated to contain 350 pM soluble aggregates (Supplementary Fig. 1). Above 10 nM total monomer there is formation of additional aggregates, so the oligomer dose increases with time. There is also no significant cell death for total monomer concentrations below 40 nM (Supplementary Fig. 2). We found that both BV2 microglial cell line and astrocytes showed a sensitized response with time and this was significant at oligomer concentrations down to 10 pM (Fig. 2), but there was no measurable response in TLR4 and Myd88 knockout cell lines (Supplementary Fig. 3). The Aβ aggregate-induced TLR4 signalling can be effectively blocked by the TLR4 antagonists RSLA and TAK-242 (Fig. 2).

### LTP deficit experiments

**Fig. 3:**
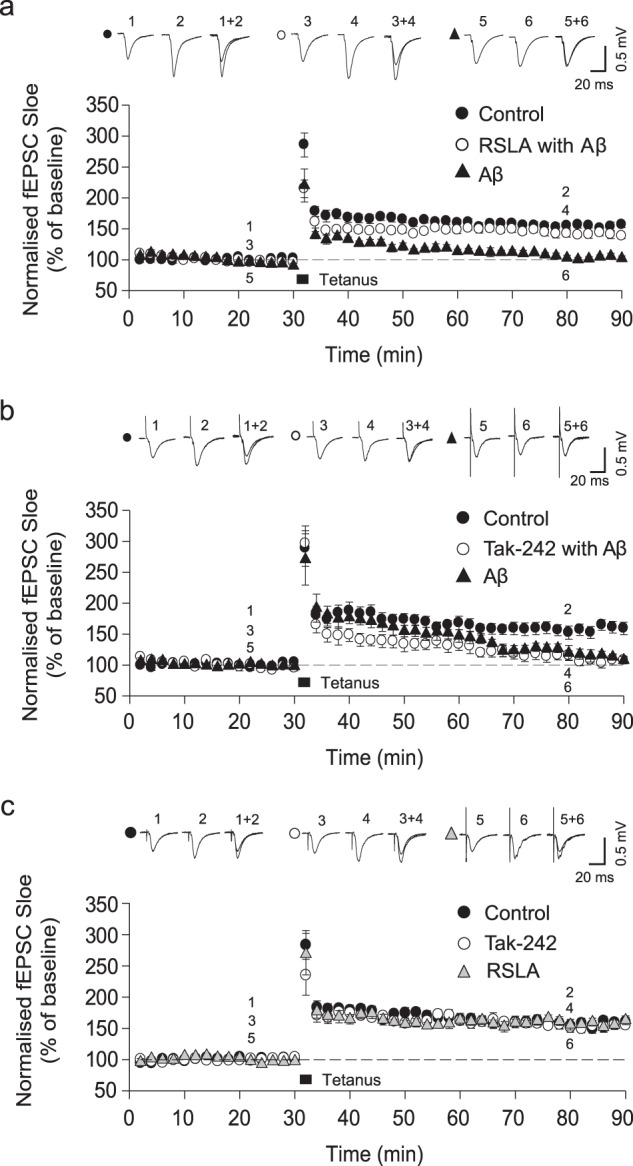
Aβ42-mediated inhibition of LTP is rescued by RSLA but not TAK-242 treatment. Top: Example traces of fEPSPs from indicated time-points. Bottom: Mean fEPSP slope shown as percentage of the normalised baseline. **a** The inhibition of LTP by application of oligomerised Aβ42 (5 nM oligomers and 500 nM total monomer) was prevented by RSLA. **b** TAK-242 had no effect on Aβ-mediated inhibition of LTP. **c** TAK-242 and RSLA had no effect on the expression of LTP compared with control. All symbols represent the mean ± sem.

Next, we tested whether the Aβ-mediated LTP deficit involved TLR4 signalling. Using rat hippocampal slices Aβ aggregates were pre-incubated for a few hours before measurement of LTP. At 500 nM total Aβ monomer (~15 nM oligomers), 100 Hz electric stimulation-induced LTP in control slices (156.3 ± 5.3%, *n* = 6), this was inhibited in slices treated with Aβ oligomers (110.9 ± 3.1%, *n* = 6; Control vs. Aβ, *p* = 0.0000228). Treatment with RSLA prevented the Aβ-mediated inhibition of LTP (146.7 ± 3.2%, *n* = 6; RSLA with Aβ vs. Aβ, *p* = 0.0000112; control vs. RSLA with Aβ, *p* = 0.155; Fig. 3a). However, TAK-242 (100 ng–1 μg/ml) did not prevent Aβ-mediated inhibition of LTP (TAK-242 with Aβ (111.6 ± 8.2%, *n* = 6); TAK-242 with Aβ vs. Aβ, *p* = 0.410; Control vs. TAK-242 with Aβ, *p* = 0.00414; Fig. 3b). TAK-242 and RSLA had no effect on the magnitude of LTP induction compared with control (control: 155.9 ± 5.7%, *n* = 7; Tak-242: 153.2 ± 6.5%, *n* = 6; RSLA: 160.1 ± 8.1%, *n* = 6; Fig. 3c). RSLA directly blocks the TLR4-binding site^17^ while TAK-242 binds an intracellular domain of TLR4^17,18^ and hence may less effectively reach its binding site in the brain slice. An Elisa assay was performed which showed increased production of TNF-α in the hippocampus on addition of Aβ aggregates (Supplementary Fig. 4). These results together show that Aβ aggregate-induced LTP deficit can be reduced by blocking TLR4 signalling.

### Neuronal cell death

**Fig. 4:**
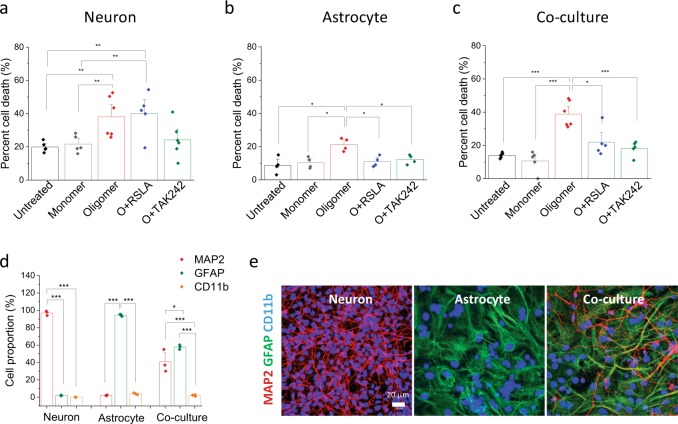
Neuronal cell death caused by Aβ42 oligomers is mediated by initiating TLR4 signalling by glial cells. Cell death assay was performed using Sytox green on a live-cell imaging platform. **a** TLR4 inhibitors (0.1 µg/ml RSA, 1 µM TAK242) did not prevent Aβ42 oligomer-induced cell death in pure neuronal culture (5 nM oligomer and 1 µM total monomer). **b**, **c** Oligomer-induced astrocyte cell death was protected by TLR4 inhibitors. **d**, **e** Composition of the co-culture assessed using MAP2 (neuronal marker) and GFAP (astrocytic marker) immunocytochemistry together with representative images of a neuronal, astrocyte and co-culture. 97 ± 1.6% are MAP2-positive cells in neuronal prep, 92.2 ± 0.1% are GFAP-positive cells in astrocytic prep. 55.3 ± 7.1% MAP2 (+) and 44.1 ± 1.3% GFAP (+) are found in co-culture prep. The proportion of CD11b-positive cells is 3.8 ± 0.4% and 1.3 ± 0.5% in the astrocytic and co-culture preparations, respectively. (*n* = 5–6 from two independent experiments, mean ± sem).

The next set of experiments were designed to determine whether neuronal cell death was mediated by the direct action of aggregates acting on neurons and how much was mediated by cytokines produced by TLR4 signalling by a paracrine/autocrine mechanism. Rat neurons (E16–17), enriched astrocytes (purchased from Science Cell) and a co-culture of astrocytes and neurons (P2–P4) were exposed to 1 μM of aggregated Aβ containing ~15 nM oligomers in the absence or presence of specific inhibitors of TLR4 signalling, RSLA and TAK-242 (Fig. 4 and Supplementary Fig. 5). Addition of aggregated Aβ to enriched astrocyte cultures induced astrocytic cell death that was prevented by TLR4 antagonists. Addition of aggregated Aβ to enriched neuronal cultures induced neuronal cell death, and this was not significantly prevented by both TLR4 antagonists. Notably, addition of aggregated Aβ to neuron and astrocyte co-cultures induced significant cell death, which was prevented by TLR4 antagonists. Since it is not possible to perform reliable immunohistochemistry on dead cells we used a method developed previously, based on measurement of the nuclei size of live cells^19^, to determine if there was a change in the proportion of surviving astrocytes and neurons in the co-culture, after treatment. The astrocytes and neurons have nuclei size in distinct size ranges with the neurons having smaller nuclei. Live cell imaging (Supplementary Fig. 6a, b) suggested that neurons were more vulnerable in the co-culture, since we observed a significant increase in the average nuclei size of the surviving cells after treatment (attributable to a greater proportion of surviving astrocytes). We confirmed this result in an independent experiment where we performed immunocytochemistry on the co-culture, before and after addition of aggregated Aβ, and found that the proportion of surviving astrocytes increased and the proportion of neurons decreased (Supplementary Fig. 6c, d), consistent with predominantly neuronal cell death. Our data suggests that although both astrocytes and neurons exhibit reduced viability on exposure to aggregated Abeta, the glial cells are less vulnerable to cell death than the neurons. Overall our experiments show that the main cause of neuronal cell death in this acute dose experiment is caused by aggregate-initiated TLR4 signalling by glial cells, astrocytes and the small fraction of microglial present in the enriched astrocyte preparation. We cannot work out the relative contribution of astrocytes and microglia to this overall response, since although only 2% of the glial are microglia, they have higher expression of TLR4.

## Discussion

Here we have shown using rat neuron and glial cells that Aβ aggregates cause LTP deficit and neuronal cell death predominantly by an autocrine/paracrine mechanism due to the production of pro-inflammatory cytokines, predominantly by TLR4 signalling by glial cells although we cannot rule out some contribution from neurons in the LTP deficit experiment. These experiments were performed with high doses of soluble aggregates but we also showed that physiological doses of aggregates over longer timespan also lead to the production of pro-inflammatory cytokines due to a sensitized response, as observed previously with alpha synuclein aggregates^19^. The concentration of TNF-α produced after several days approaches that observed in the CSF of individuals with AD, 400 pg/ml^20^ and the concentration produced by larger doses of aggregates in the LTP-deficit experiments, 250 pg/ml.

There is a body of work that supports inflammation and aggregation occur concurrently in the development of the pathology of AD in both animal models and in humans, suggesting positive feedback between aggregation and inflammation^21^. Furthermore, acute doses of aggregates can lead to cognitive dysfunction^22^ mediated by the TNF receptor^23^ and involving activation of P38 MAP kinase^24^. However, the relevance of these observations to AD, which takes decades to develop, and the mechanism by which cognitive dysfunction occurs in AD has not been established. Our data suggests that sensitized aggregate-induced inflammation over time leads to LTP deficit and neuronal death, via TLR4 signalling, providing a molecular basis for the memory loss observed in the progression of AD, supported by the previous in vivo studies^22,23^. While our experiments suggest that the TLR4 signalling is predominantly from glial cells we cannot rule out a contribution from neurons and in addition TLR4 expression has been shown to increase as neurons age^25^, suggesting that the neuronal contribution may increase with age in AD. Importantly our results point to an autocrine/paracrine mechanism due to secreted pro-inflammatory cytokines rather than direct binding of aggregates to receptors on neurons. In terms of the relevance of our finding to disease, we have recently found that the CSF from AD patients is significantly more inflammatory than control CSF when applied to BV2 cells over 5 days^26^. This shows that increased low levels of inflammatory aggregates occurs with the development of AD and in combination with the results in this paper that aggregate-induced inflammation could contribute significantly to the memory loss observed in these individuals. This inflammatory response is mediated by Aβ containing aggregates in the CSF, via TLR4 signalling, and correlates with the presence of protofibrils. Our recent work also shows that synthetic Aβ aggregates exist in a range of different size and structures with the longer protofibrils being the inflammatory species^11^. Selectively targeting these protofibrils rather than the fibrils, smaller aggregates or monomer is challenging, so that blocking sensitized TLR4 signalling seems an attractive alternative therapeutic strategy in AD. Memory loss is an early symptom of AD and continues throughout the disease, so this strategy could be used at all stages of disease and TLR4 antagonists could potentially be given intermittently, given the nature of the priming response observed in this work.

## Methods

### Protein preparation

The Aβ was purchased from American peptide (Aβ 1–42, 1.0 mg). 1 mg of amyloid pure peptide was dissolved in 100% 1, 1, 1, 3, 3, 3-Hexafluoro-2-propanol (HFIP) to a concentration of 2 mg/ml and incubated at room temperature until a clear solution was formed. The solution was then dried under a nitrogen stream before dissolved once more in HFIP and sonicated. 100 μl of solution was then aliquoted into an eppendorf and stored at −80 °C. When required the solution was thawed and left open in a fume hood overnight to evaporate the HFIP, leaving a peptide pellet. The peptide was dissolved in 10 μl of 100%DMSO and then transferred to a new eppendorf with DMEM.

### Protein aggregation

Aβ oligomers were prepared by first diluting in DMEM buffer (DMEM + 1% FCS + 2 mM l-glutamine). The aggregation mixture was incubated for 6 h at 37 °C with constant shaking of 200 r.p.m (New Brunswick Scientific Innova 43, 25 mm orbital diameter) and centrifuged for 10 min at 14,200 r.p.m. to remove any fibrillar pellets. Aβ fibrils were formed by aggregation for 60 h. Aggregated Aβ was then stored at 4 °C until incubated with cells. Using ThT assays protein oligomers and fibrils were found to remain stable for 1 week after removal from the shaking incubator however aggregates were always used within 24 h.

### ThT assay

The time course of the aggregation was monitored using thioflavin-T (ThT) assays. ThT (Sigma-Aldrich) stocks were prepared in DMSO (Sigma-Aldrich) and diluted into pre-filtered PBS (0.02 μm filter, Whatman) to a final concentration of ~100 μM. Aβ was added to 1 ml of ThT solutions and binding monitored by exciting the sample at 440 nm and recording the emission fluorescence spectrum from 460 to 560 nm (slit width 5 nm). Measurements were carried out on a Cary Eclipse spectrofluorometer with a Peltier-controlled holder (Varian, Mulgrave, Australia).

### ThT imaging

ThT imaging utilised a method previously described^27^. Briefly glass cover-slides (VWR international, 20 × 20 mm) were cleaned using an argon plasma cleaner (PDC-002, Harrick Plasma) for at least 1 h to remove any residual that fluoresce. 50 µL of poly-l-lysine (70,000–150,000 molecular weight, Sigma-Aldrich) was added to the cover slides and incubated for 1 h before being gently washed with filtered PBS. Imaging was performed on a custom-built total internal reflection fluorescence microscope.

### Cell culture

The BV2 cell lines were derived from immortalized murine neonatal microglia. They were grown in Dulbecco’s modified Eagle’s (DMEM) supplemented with 10% foetal bovine serum and 1% l-glutamine (Life Technologies) and incubated at 37 °C in a humidified atmosphere of 5% CO_2_ and 95% air, until ~1.6 × 10^6^ cell/ml. Immortalized MyD88^−/−^, TLR2^−/−^ and TLR4^−/−^ murine cells had previously been generated^19^ and grown from frozen stock samples under same conditions as BV2. Astrocyte cells were from a rat mixed glial preparation, cultured for 14 days with DMEM medium supplemented with 10% foetal bovine serum and 1% l-glutamine in a humidified atmosphere containing 5% CO_2_ at 37 °C. This protocol minimizes the microglial contamination to <2%^28^. Astrocytes were cultured to 70% confluence. For long duration experiments the media was exchanged every 24 h.

### ELISA assays

To determine cumulative TNF-α and Il-1β production, supernatants were obtained after incubation with the Aβ over viable time frames and stored at −80 °C until analysed. TNF-α, and Il-1β were analysed using the Duoset® enzyme-linked immunosorbent assay (ELISA) development system (R&D Systems, Abingdon, Oxfordshire, UK).

### Animals

Electrophysiology experiments were conducted in accordance with the UK Animals Scientific Procedures Act, 1986. Male Wistar rats (Charles River, UK) were used to prepare acute hippocampal slices (4- to 5-week-old rats).

### Electrophysiology

Brains were quickly removed into ice-cold artificial cerebrospinal fluid (ACSF; 124 mM NaCl, 3 mM KCl, 26 mM NaHCO_3_, 1.25 mM NaH_2_PO_4_, 2 mM CaCl_2_, 1 mM MgSO_4_, 10 mM d-glucose, carbogenated with 95% O_2_/5% CO_2_). Transverse hippocampal slices (400 µm) were cut using a Mcllwain tissue chopper (Mickle Laboratory Engineering) and allowed to recover in a submersion-type bath filled with ACSF for at least 60 min. Before recording, hippocampal slices were incubated in ACSF with pharmacological compounds at room temperature. Evoked field excitatory postsynaptic potentials (fEPSPs) were recorded in the CA1 region using glass electrodes containing NaCl (3 M). Twisted-tungsten wire stimulating electrodes were positioned in the CA2 region (Schaffer collateral pathway) and subiculum, and electric stimuli were delivered alternatively to the two electrodes (0.066 Hz). After 30 min of stable baseline, high-frequency tetanic stimulation (2 × 100 pulses; 100 Hz, 30 s interval) was used as the LTP induction protocol. Long-term synaptic plasticity was gauged as the change of fEPSP slope relative to the preconditioning baseline. Both control and experimental recordings were collected using slices prepared from the same animal. Data were captured and analysed using LTP114j software. Data are expressed relative to a normalized baseline. For analyses, the baseline was defined as five time-points before tetanic stimulation and the post-conditioning time was set at 75–80 min following recording commencement. The difference between baseline and post-conditioning time-points was expressed as a percentage of baseline ± standard error of the mean (SEM), and was used to make comparisons between treatment groups. Statistical significance of observed effects between groups was analysed using unpaired *t*-tests.

### Aβ preparation for LTP experiments

The Aβ (1–42) peptide (Stratech, A-1163, 0.5 mg) was initially dissolved at a concentration of 1 mg/ml in 100% (HFIP [Sigma-Aldrich]). This solution was incubated at room temperature for 1 h with vortexing every 10 min. Next, the solution was sonicated for 10 min in a water bath sonicator and then dried under a light stream of nitrogen gas. DMSO was added to the peptide, which was incubated at room temperature for 10 min with gentle mixing. Finally, this solution was aliquoted and stored at −80 °C. For a working solution, D-PBS (Invitrogen, UK) was added to the peptide stock solution and incubated for 2 h at room temperature for peptide oligomerisation. 500 nM oligomerised amyloid-beta was applied to hippocampal slices in ACSF for 2 h. Previous experiments show this protocol results in a solution containing 1–5 nM oligomers smaller than 10-mers^15^.

### Rodent neuron and astrocyte culture

Cultures of cortical neurons and the co-culture of neuron and astrocyte were prepared as described previously^19^. Briefly, cortices of brain from either embryos (E16–17) or postnatal pups (day 2–4) of Sprague-Dawley (UCL breeding colony) were collected for neuron and co-culture preparations, respectively. The tissue was digested with EDTA–trypsin for 15 min and washed before collecting pellets in complete neurobasal medium (Neurobasal media supplemented with B27, 2 mM Glutamax and 50 I.U./ml Penicillin/50 μg/ml Streptomycin). Approximately 50,000 cells for 96-well plates (Falcon/Corning Cat. no.: 353219) and 100,000 cells for u-slide eight-well slide ibidi chamber (Thistle Scientific Ltd, Cat. no.: IB-80826) were plated. Cells were used at 12–14 days in vitro. Cortical astrocytes were purchased from Caltag Med Systems (Science Cell, Cat. no.: R1800) which was derived from postnatal day 2 rat cortex. 7–10 times sub-culture of cortical astrocytes were carried out until use. Cells were cultured in rodent astrocyte medium (Caltag Medsystem, Cat. no.: 1831). All cells were maintained in incubator at 37 °C (5% CO_2_).

### Cell death assay

Cells were pre-treated with either 0.1 μg RSLA or 1 μM TAK-242 30 min prior to oligomer treatment. Cells were incubated with soluble aggregates overnight and cell death was detected using Sytox green (Molecular Probes). Cells were loaded with 0.5 μM Sytox green in HEPES balanced HBSS (pH adjusted at 7.4 with NaOH) for 15 min. High-throughput images were acquired using an Opera Phenix High-Content Screening System (PerkinElmer). Sytox green staining was imaged by 488 and 405 nm laser for Hoechst-stained nuclei (17–22 fields of images were taken per wells). The percentage cell death was quantified by the ratio between the number of Sytox green-positive cells and the total number of Hoechst-expressing cells per image. The number of fluorescent cells was determined using the multi-wavelength cell scoring module of Columbus Studio™ Cell Analysis Software and each experiment utilised the same threshold settings (e.g. intensity and size). Nuclei size of live cells (Sytox negative) was automatically acquired using the same analysis software first in the neuron only culture, and the enriched astrocyte culture. These size ranges were then used to determine the culture composition of the co-culture before and after treatment with Abeta aggregates.

### Immunohistochemistry

Cells were fixed in 4% paraformaldehyde and permeabilized with 0.2 Triton-X 100. Non-specific binding was blocked using 5% BSA. Cells were incubated with primary antibodies for 1 h (MAP2; ab11267, GFAP; ab4674, CD11b; ab133357) at room temperature and washed three times. Secondary antibodies (donkey anti rabbit 488; ab150073, donkey anti-mouse; ab150110, goat anti-chicken 647; ab150171) were incubated for 1 h at room temperature. Cells were washed three times and Hoechst was added in the second wash. Cells were imaged with ProLong Diamond Antifade Mountant (Thermo Fisher Scientific).

### Statistics and reproducibility

For live cell imaging, there were total two independent experiment which include different animals, different cell batch purchased separately and cell preparation. Each set of independent experiments consists of three wells per condition and the figures represent data polled from two independent experiments. One-way ANOVA with Bonferroni or Tukey correction was used to test statistical significance. One-way ANOVA with post hoc test was used to test statistical significance in the production of TNF-α in the slice experiments.

### Reporting summary

Further information on research design is available in the Nature Research Reporting Summary linked to this article.

## Supplementary information

Supplementary Information

Description of Additional Supplementary Files

Supplementary Data 1

Supplementary Data 2

Supplementary Data 3

Reporting Summary

## Data Availability

Source data underlying Figs. 1 and 2 are in the Supplementary Data 1 file, Fig. 4a–c in Supplementary Data 2 file and Supplementary Fig. 4d in Supplementary Data File 3. All other data are available upon request from the corresponding author.
