# Supplementary material for: Beta amyloid aggregates induce sensitised TLR4 signalling causing long-term potentiation deficit and rat neuronal cell death
Source: Commun Biol. 2020 Feb 18;3:79. doi: 10.1038/s42003-020-0792-9 (PMC7028984; doi:10.1038/s42003-020-0792-9)
Supplement: Supplementary file 1 — Supplementary Information [file 42003_2020_792_MOESM1_ESM.pdf]

Supplementary Information

a

b

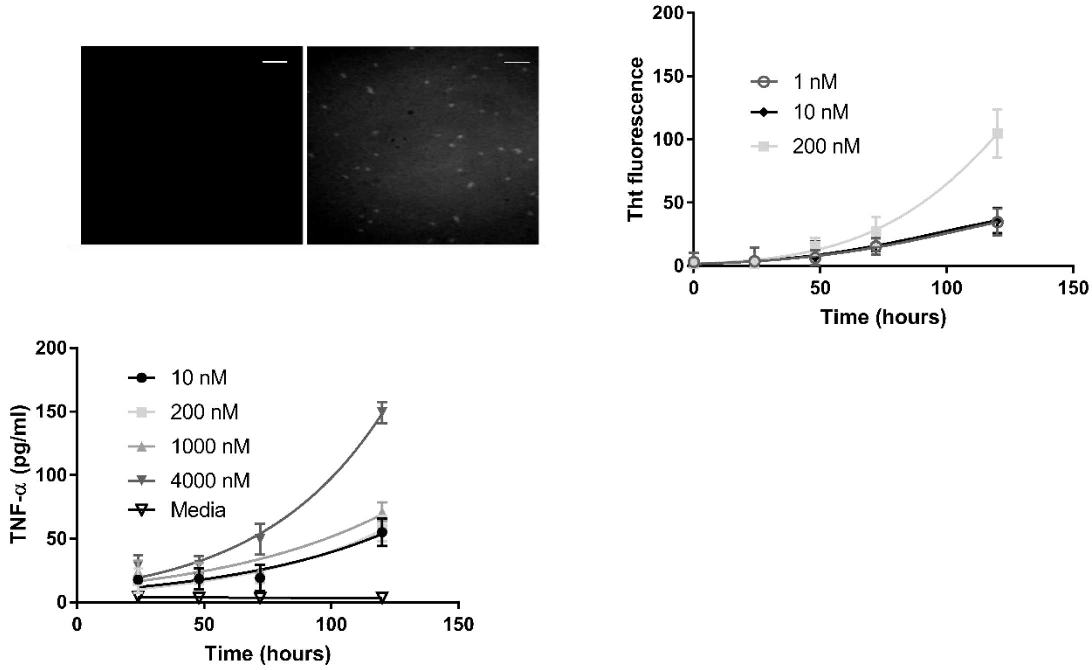

Supplementary Figure 1. Formation of soluble Abeta42 aggregates from monomers over time. A) TIRF image of fluorescent ThT bound Aβ (200 nM total monomer) at 0 hours and 120 hours (left and right panel respectively) after incubation with BV2 microglia cells. Scale bar represents 5 µm. B) ThT assay of momomeric Aβ (1 nM, 10 nM and 200 nM total monomer) (n=3, sem). C) Pro-inflammatory response, measured by TNF-α production in BV2 cells, after incubation of the cells with Aβ monomers (n=3, mean +/- sem).

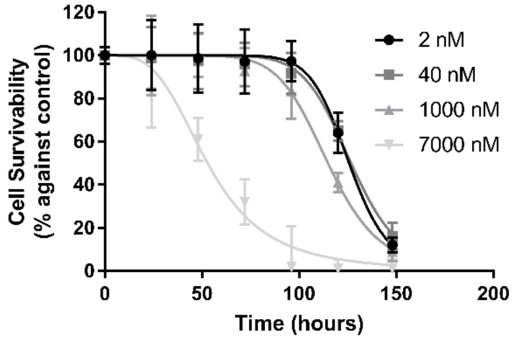

Supplementary Figure 2. BV2 microglia cell survivability after incubation with different concentrations of soluble Abeta42 aggregates and monomer. BV2 cells incubated for 144 hours with soluble aggregates at a total monomer concentration of 2 nM, 40 nM, 1000 nM and 7000 nM (initial oligomer concentration 0.01 nM, 0.2nM , 5nM and 35 nM respectively) with buffer exchanged every 24 hours. Cell count were taken every 24 hours (n=3, mean +/- sem).

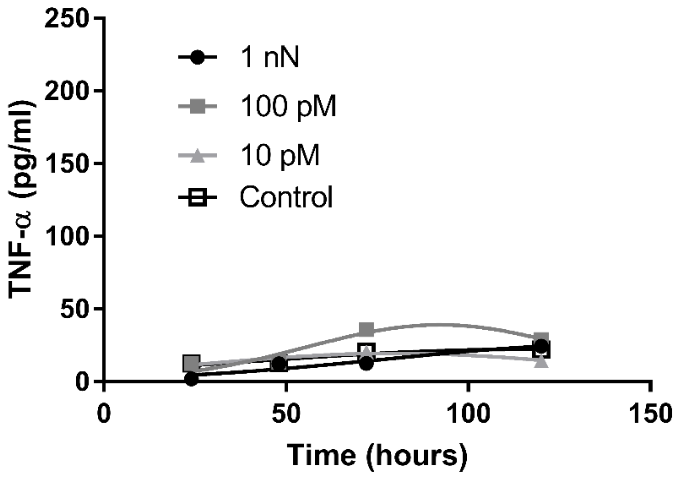

Supplementary Figure 3. Pro-inflammatory response to different concentration of Abeta42 oligomers with monomer measured in TLR4 knock-out macrophages (initial oligomer concentration 1 nM, 100 pM and 10 pM and total monomer concentration 0.2, 0.02 and 0.002 µM respectively)(n=3, mean +/- sem).

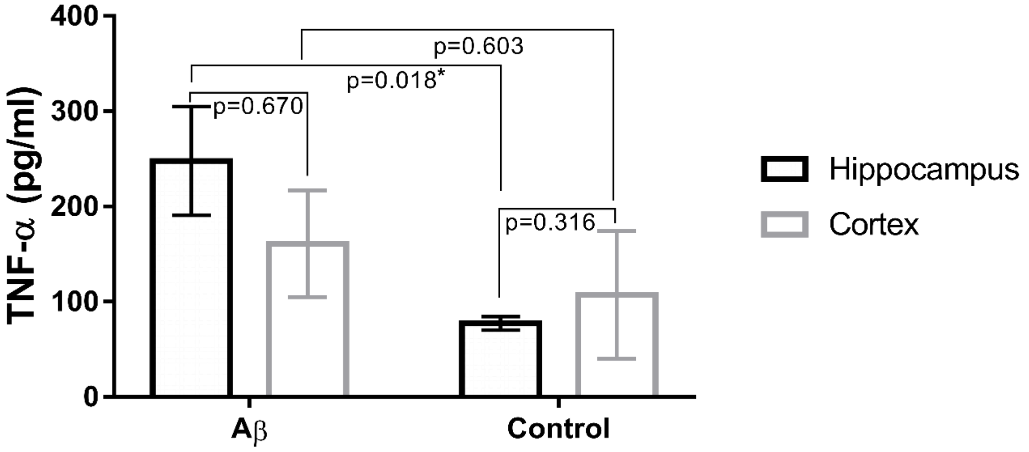

Supplementary Figure 4. TNF-α production in different regions of the brain slice after LTP deficit experiments (n=6, mean +/- sem).

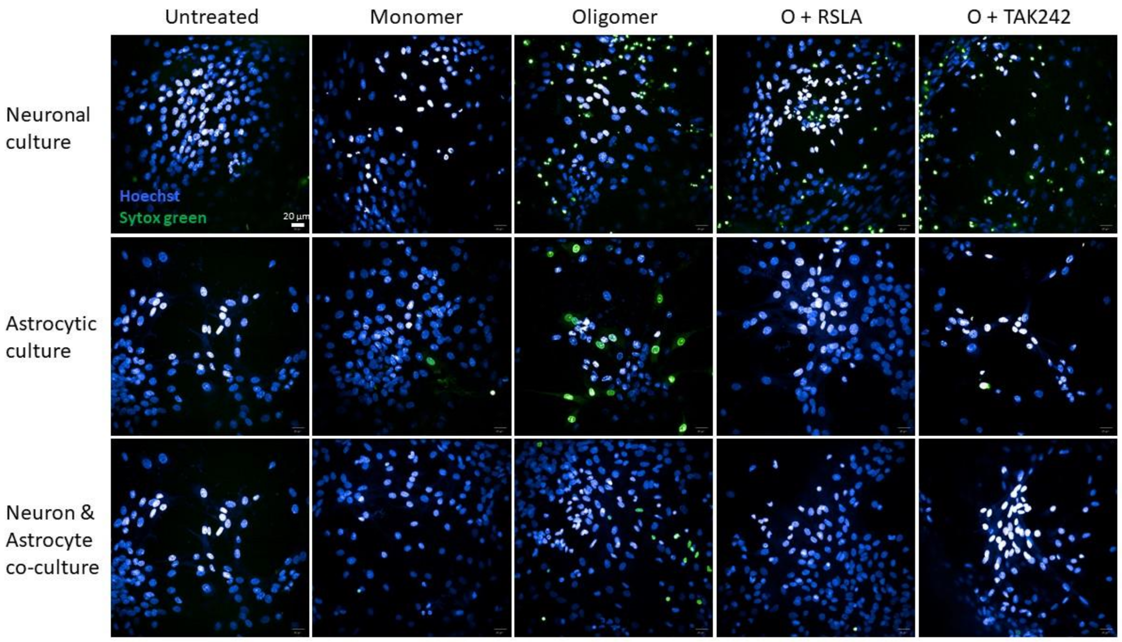

Supplementary Figure 5. Representative images for Figure 3 A, B & C. Cell death was measured using Sytox green. High-throughput live cell imaging was performed using a 40x objective. Scale bar = 20?m.

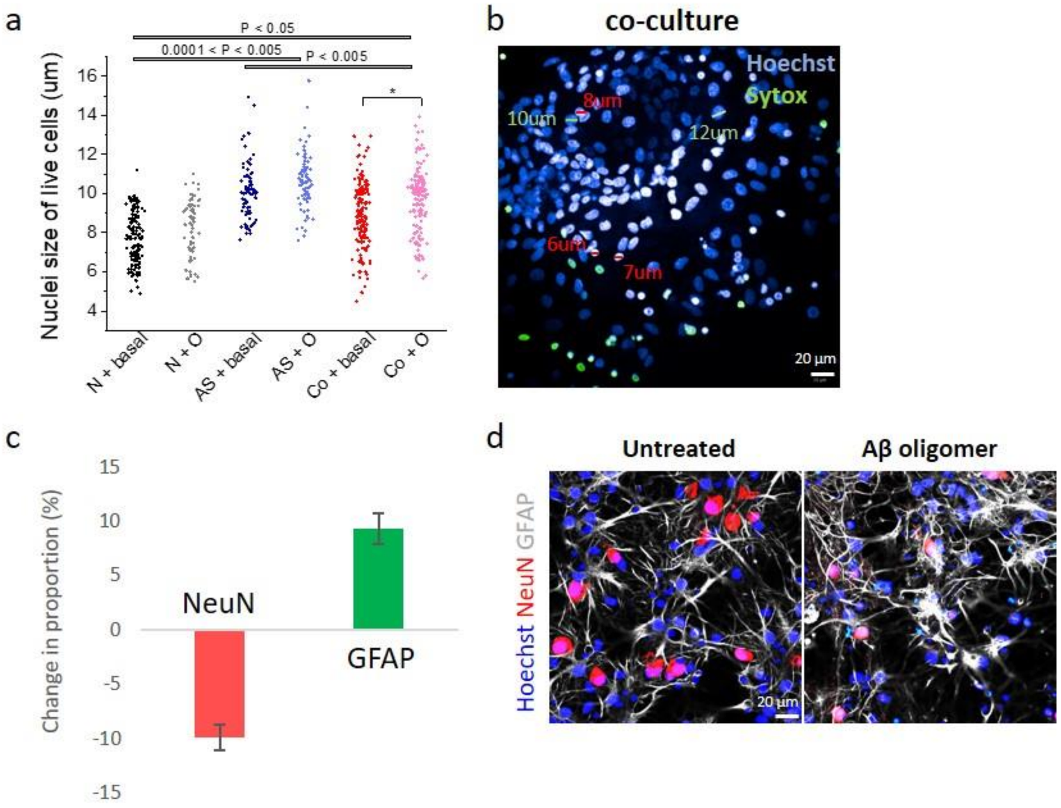

Supplementary Figure 6. Neurons are more affected by Aβ42 oligomer than astrocytes. a) Nuclei size of live cells (Hoechst positive, Sytox negative) at basal levels and post oligomer treatment (5 nM oligomer and 1 µM total monomer) was measured using a live cell imaging platform. Live neurons have a nuclei size range of 4 -10 µm and live astrocytes have larger nuclei range of 8 - 12 µm size. In co-culture prep, the population of nuclei size is distributed approximately from 6 to 12 µm. Post oligomer treatment, the population shifts to cells with larger nuclei, suggesting astrocytic survival (sem, n = 64 - 164 cells). b) Representative image showing the nuclei size of surviving cells in the neuron and astrocyte co-culture after treatment with Aβ oligomers. c) The proportion of neurons and astrocytes in the co-culture before and after treatment with Aβ oligomers was measured in an independent experiment using immunostaining (sem, n = 4 images). Before treatment 34% of the live cells were neurons and 62% were astrocytes. Treatment with Aβ oligomers induced 15.3 ± 1.4 % cell death (measured by sytox green). There is an increase in the astrocyte proportion (measured by GFAP immunocytochemistry) while the proportion of neurons (measured by NeuN immunocytochemistry) decreases. d) Representative images showing that the neuronal proportion (NeuN positive cells) is lower than untreated condition in the co-culture prep. Scale bar = 20 µm.
