# Supplementary material for: Beta amyloid aggregates induce sensitised TLR4 signalling causing long-term potentiation deficit and rat neuronal cell death
Source: Commun Biol. 2020 Feb 18;3:79. doi: 10.1038/s42003-020-0792-9 (PMC7028984; doi:10.1038/s42003-020-0792-9)
Supplement: Supplementary file 2 — Description of Additional Supplementary Files [file 42003_2020_792_MOESM2_ESM.pdf]

Descriptions of additional supplementary files

Supplementary Data File 1 contains the data underlying Figures 1.

Supplementary Data File 2 contains the data underlying Figure 4 a), b) and c .

Supplementary Data File 3 contains the data underlying Supplementary Figure 4d).
